# Immune response evaluation in *Balb/c* mice after crude extract of *Anisakis typica* sensitization

**DOI:** 10.14202/vetworld.2019.1529-1534

**Published:** 2019-10-04

**Authors:** Linda Haryadi, Eddy Suprayitno, Aulanni’am Aulanni’am, Anik Martinah Hariati

**Affiliations:** 1 Doctoral Program, Faculty of Fisheries and Marine Sciences, Brawijaya University, Jalan Veteran, Malang 65145, East Java, Indonesia; 2 Fish Quarantine and Inspection Agency of Kupang, Jalan Ade Irma No 6 Walikota, Kupang 85228, East Nusa Tenggara, Indonesia; 3 Department of Food Processing Technology, Faculty of Fisheries and Marine Sciences, Brawijaya University, Jalan Veteran, Malang 65145, East Java, Indonesia; 4 Department of Biochemistry, Faculty of Sciences, Brawijaya University, Jalan Veteran, Malang 65145, East Java, Indonesia; 5 Department of Aquaculture, Faculty of Fisheries and Marine Sciences, Brawijaya University, Jalan Veteran, Malang 65145, East Java, Indonesia.

**Keywords:** allergy, dendritic cells, inflammation, nematode, regulatory T cells

## Abstract

**Background and Aim:**

*Anisakis* is a global challenge for a fish product which may lead to a decrease in economic value and consumers’ preference. Skipjack (*Katsuwonus pelamis*) in Kupang, Nusa Tenggara Timur, Indonesia, have important economic value for local fisheries. *Anisakis typica* is one of the *Anisakis* species which potent to induce an allergic reaction. However, the study about *A. typica* involved in the dendritic cells (DCs), T helper 1 (Th_1_), T helper 2 (Th_2_), and regulatory T cells (Tregs) is still limited. This study aimed to analyze the dynamic changed of the immune system including DCs, CD4^+^ T cells, and Tregs after 1 week of *A. typica* sensitization.

**Materials and Methods:**

Twenty-four male *Balb/C* mice were randomly divided into four groups (n=6), mice treated with crude *A. typica* extract (CAE) 50, 75, and 100 mg/kg BW, respectively. CAE was given orally per day for a week. At the end of the experiment, the animals were sacrificed and the spleen was collected. DCs were labeled as CD11c^+^ interleukin-6^+^ (IL-6^+^); CD4^+^ T cells were distinguished as Th_1_ (CD4^+^ interferon-γ^+^ [IFN-γ^+^]) and Th_2_ (CD4^+^ IL-4^+^ and CD4^+^ IL-5^+^); Tregs were labeled as CD4^+^CD25^+^CD62L^+^. The expression of each cell was determined by flow cytometry.

**Results:**

Our result described that CAE elicits CD11c^+^ IL-6^+^, CD4^+^ IFN-γ^+^, CD4^+^ IL-4^+^, and CD4^+^ IL-5^+^ and reduces CD4^+^CD25^+^CD62L^+^ significantly (p<0.05) in dose-dependent manner in mice after *A. typica* infection.

**Conclusion:**

The Th_1_/Th_2_ ratio after *A. typica* crude extract treatment exhibits a mixed pattern rather than the classical model allergy to food antigens. Our study is expected as a basic understanding of the changes in immune response after *A. typic* a infection.

## Introduction

Food safety and food security due to food-borne infections are gaining interest in the past decade [1,2]. Anisakiasis, the zoonotic disease caused by nematode larvae of the genus *Anisakis* is considered as one of the most important biohazards in the fish products [3]. The previous study reported that *Anisakis* spp. were found in commercially fish, particularly anchovies (*Engraulis encrasicolus*), sardines (*Sardina pilchardus*), European hake (*Merluccius merluccius*), whiting (*Merlangius merlangus*), chub mackerel (*Scomber japonicus*), and Atlantic bluefin tuna (*Thunnus thynnus*) [4].

*Anisakis* spp. have a complex life cycle and reach maturation in the third stage (L3). Marine mammals are a final host to complete its life cycle [5]. A human can accidentally be infected by *Anisakis* after consumed the raw or half-cooked fish meats, which is strongly associated with acute gastrointestinal (GI) symptoms [6] and allergen reaction [7]. Both live and death larva of *Anisakis* could induce the allergic reaction due to its thermal- and pepsin-resistant properties [5,8]. Interestingly, the simple prepared crude extract of *Anisakis* is enough to induce the allergic reaction [9].

The allergic reaction due to *Anisakis* has been reported to elicit the host immune response which is characterized by T helper 2 (Th_2_) response predominantly by secreting cytokines such as interleukin-4 (IL-4) and IL-5 [10]. Furthermore, T helper 1 (Th_1_) maturation by dendritic cells (DCs) was suppressed by regulatory T cells [11] which assist Th_2_ polarization during helminth infection. This regulatory network results in the decrease of interferon-γ (IFN-γ), the cytokine which secreted by Th_1_ [12]. In contrast, the previous study reported that *Anisakis* allergy exhibits a mixed Th_1_/Th_2_ pattern [13]. Meanwhile, prolonged nematode infection may lead to chronic infection predominantly by Th_1_ [14].

Nowadays, there are nine *Anisakis* species which have been confirmed [15]. Among them, *Anisakis simplex* sensu stricto (s.s) and *Anisakis pegreffii* are the best known caused the allergic reaction and other health problems [16]. However, as far as our knowledge, there is little information about allergen reaction which involved immune cells such as DCs, Th_1_, Th_2_, and regulatory T cells (Tregs) caused by *Anisakis typica*. *A. typica* have been found parasitizes Delphinidae, Phocoenidae, and Pontoporidae in Atlantic and Indian Oceans and in the Eastern Mediterranean Sea. Surprisingly, *A. typica* also found in Australian and Indonesia [17]. This finding increases the potential risk for anisakiasis frequency in Southeast Asia, mainly in Indonesia.

This study aimed to evaluate the immune response underlying host after *A. typica* infection. The present study is expected as a basic understanding of *A. typica* accidental infection due to marine products and, subsequently, develops the intervention strategies.

## Materials and Methods

### Ethical approval

This study was approved by the Ethical Committee of Brawijaya University (approval number 938-KEP-UB).

### Animals

Male *Balb/C* mice aged 5 weeks were supplied from the Integrated Research and Testing Laboratory-Unit IV, Gadjah Mada University. Mice were housed in plastic cages for a period of acclimatization. Mice were given food and water *ad libitum* and maintained at room temperature with a 12 h light/dark cycle.

### Crude *A. typica* preparation

Skipjack (*Katsuwonus pelamis*) were purchased from the traditional market in Kupang, Nusa Tenggara Timur, Indonesia. *A. typica* was manually harvested from the abdominal cavity of skipjack which naturally parasitized by *A. typica*. *A. typica* was identified by polymerase chain reaction according to Soewarlan *et al*. [18] using NC5 (forward; 5’-GTAGGTGAACCTGCGGAAGATCATT-3’) and NC2 (reverse: 5’-TTAGTTTCTTTTCCTCCGCT-3’) primer (data not shown). *A. typica* washed with distilled water then stored at 4°C. The crude extract of *A. typica* was made by crushed *A. typica* using porcelain mortar and pestle at 4°C. The protein content of milled *A. typica* then measured using nanodrop spectrophotometer (ND1000). The protein content then considered as a standard to determine the dosage given to animals.

### Experimental design

Twenty-four male *Balb/C* mice weight 25 g were randomly and equally divided into four groups (n=6): Normal (unsensitized mice) and mice treated with crude *Anisakis* extract (CAE) 50, 75, and 100 mg/kg BW, respectively. Mice were intragastrically challenged per day for 7 days consecutively with CAE, except the normal group. At the 8^th^ day, mice were anesthetized through intraperitoneal injection the combination of ketamine and xylazine (90 mg/kg and 10 mg/kg, respectively) [19] followed by euthanized by cervical dislocation. The spleen was collected and washed 3 times in sterile phosphate-buffered saline (PBS) then crushed into single-cell suspensions. Single-cell suspensions then added with PBS until reached 10 mL and centrifuged at 2500 rpm for 5 min at 10°C. The supernatant then discarded and the pellet was homogenized with 1 mL PBS [20]. Homogenates then divided into several 1.5 mL tubes according to the staining used then centrifuged at 2500 rpm for 5 min at 10°C.

### Cell staining and flow cytometry analysis

The supernatant then discarded and the pellet stained according to: (1) To determine Th cells, cells surface was stained with fluorescein isothiocyanate (FITC) anti-mouse CD4 (BioLegend, clone: GK1.5), (2) regulatory T cells were identified by a combination of cell surface antibodies of FITC anti-mouse CD4 (BioLegend, clone: GK1.5), phycoerythrin (PE) anti-mouse CD25 (BioLegend, clone: 3C7), and PE-Cyanine5 (PE-Cy5) anti-mouse CD62L (BioLegend, clone: MEL-14), and (3) DCs were identified by cell surface antibodies FITC anti-mouse CD11c (BioLegend, clone: N418). Briefly, 50 μL of Cytofix/Cytoperm Buffer (BioLegend, cat no: 420801) was resuspended in pellet for 20 min in dark condition at 4°C. Then, homogenates were added with 300 μL wash-perm (BioLegend, cat. no.: 421002) and centrifuged at 2500 rpm at 4°C for 5 min. Supernatant was discarded, and the pellet was stained with intracellular staining (50 μL) of PE anti-mouse IL-6 (BioLegend, clone: MP5-20F3) which combined with CD11c. Pellet which previously stained with anti mouse-CD4 were stained with PE-Cy7 anti-mouse IL-4 (BioLegend, clone: 11B11) and PE-anti-mouse IL-5 (BioLegend, clone: TRFK5) to identified T_h_2 Besides, pellet which previously stained with anti mouse-CD4 were stained with PE anti-mouse IFN-γ (BioLegend, clone: XMG1.2) to identified T_h_1. Data were obtained using FACSCalibur^™^ (BD Biosciences, San Jose, CA, USA). A total of 10,000 cell events were collected for each sample. The cell suspensions for each sample were collected immediately with low or medium flow rate. The single-cell populations were gated according to the staining used for further analysis. Data analysis was conducted using software BD CellQuest Pro^™^ (BD Biosciences, San Jose, CA, USA).

### Statistical analysis

Statistical analyses were performed using Microsoft Excel 2016. All data were expressed as mean±standard deviation. p<0.05 was determined using one-way ANOVA followed by Duncan’s multiple range test.

## Results

### Anisakis treatment elicits DCs activation and decline regulatory T cell

**Figure-1:**
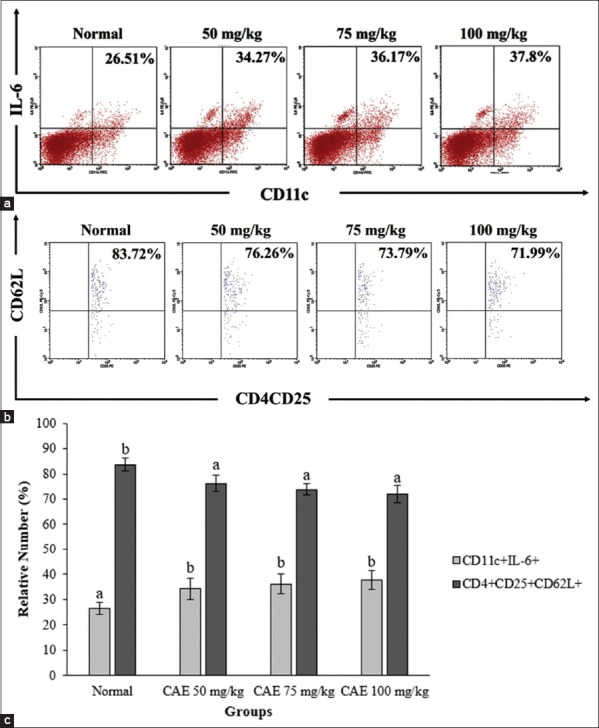
Flow cytometry analysis exhibited dendritic cells and regulatory T cells. (a) CD11c^+^ interleukin-6^+^ (IL-6^+^) expressions on splenocytes, (b) CD4^+^CD25^+^CD62L^+^ expressions on splenocytes, and (c) CD11c^+^ IL-6^+^ and CD4^+^CD25^+^CD62L^+^ expressions were represented as mean±standard deviation (n=6 for each group). The different letter on the chart was considered significantly different for each group (p<0.05) and vice versa based on Duncan’s multiple range test. CAE=Crude *Anisakis* extract.

Dose-dependent increase DCs in mice treated with *A. typica* crude extract (Figure-1a). The degree of CD11c^+^ IL-6^+^ was increasing significantly (p<0.05) in treated mice compared with normal mice (Figure-1c). In contrast, regulatory T cells expression (CD4^+^CD25^+^CD62L^+^) was decline significantly (p<0.05) in treated mice compared with normal mice (Figure-1b). The increase of CD11c^+^ IL-6^+^ and the decrease of CD4^+^CD25^+^CD62L^+^ showed a dose-dependent manner after *A. typica* crude extract treatment (Figure-1c).

### *Anisakis* treatment enhance Th_1_/Th_2_ ratio in sensitized mice

**Figure-2:**
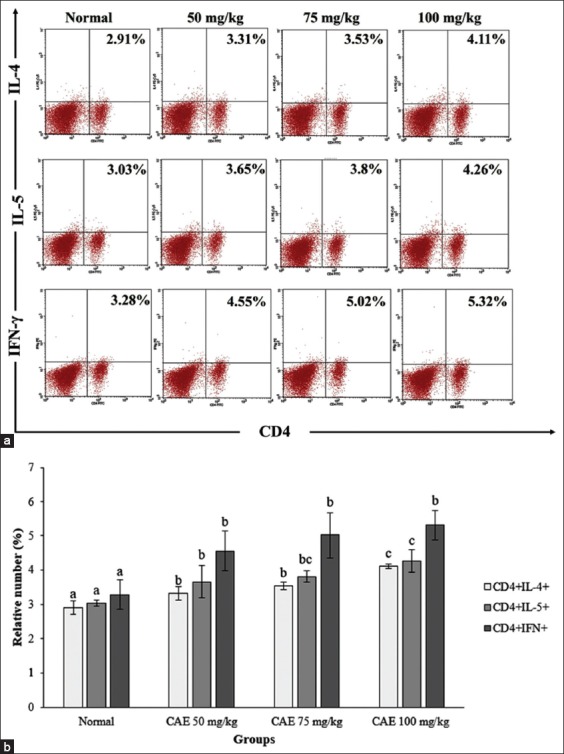
Flow cytometry analysis exhibited T helper 1 and T helper 2 cells. (a) CD4^+^ interleukin-4^+^ (IL-4^+^), CD4^+^ IL-5^+^, and CD4^+^ interferon-γ^+^ (IFN-γ^+^) expressions on splenocytes and (b) CD4^+^ IL-4^+^, CD4^+^ IL-5^+^, and CD4^+^ IFN-γ^+^ expressions were represented as mean±standard deviation (n=6 for each group). The different letter on the chart was considered significantly different for each group (p<0.05) and vice versa based on Duncan’s multiple range test. CAE=Crude *Anisakis* extract.

To determine how the immune response shifted after *A. typica* crude extract treatment, Th_1_- and Th_2_-related cytokine productions were analyzed in the spleen (Figure-2a). Our result suggests that Th_2_ (CD4^+^IL-4^+^ and CD4^+^IL-5^+^) was increased significantly (p<0.05) after *A. typica* crude extract treatment. In addition, CD4^+^ IFN-γ^+^ (Th_1_) also increases significantly (p<0.05) after *A. typica* crude extract treatment. Interestingly, CD4^+^ IL-4^+^ and CD4^+^ IL-5^+^ expression were higher in a low dose of *A. typica* crude extract (Figure-2b). Surprisingly, our present study resulting in a higher expression of CD4^+^ IFN-γ^+^ compared to CD4^+^ IL-4^+^ and CD4^+^ IL-5^+^ expression.

## Discussion

Marine food consumption, especially fish, is quite popular globally due to its nutritional content. *Anisakis* is one of the important biohazards in fishery products which may lead to a rejection by consumers and economic losses to the fish industry [17,21]. Nowadays, among nine *Anisakis* species, *A. simplex* (s.s) and *Anisakis* *pegreffii* are best known for causing infection in human. However, there is little information about *A. typica* causing infection. *A. typica ­* challenge in our research would greatly improve knowledge of anisakiasis besides *A. simplex* (s.s) and *A. pegreffii* epidemiology.

Our result suggests that the expression of CD11c^+^ (DCs) was increased after *A. typica* treatment. DCs have a responsibility to present antigen then elicited immune response during parasite infection. Macrophage, other antigen-presenting cells have reported to secrete IL-6 through toll-like receptors activation and elicit Th_2_ polarization after antigens, native carbohydrates derived from metacestode larvae parasites sensitization [22]. These results are in line with our study which suggests that *A. typica* challenge induces DCs maturation to secrete IL-6 as a pro-inflammatory cytokine. Another study reported that *in vitro* crude extract *A. pegreffii* elicits DCs to develop immune response by increase CCL3, CXCL4, CCL4, and granulocyte-macrophage colony-stimulating factor. DCs maturation in lymph node provokes pro-inflammatory IL-6 secretion and participates in Th_2_ differentiation [23].

Tregs are known to work synchronize with Th_2_ in the early phase of infection through TGF-β signaling. Furthermore, Tregs elicit Th_2_ response counter worm infection [24] by attracting eosinophils, mast cells, basophils, and production of IgE [25]. Our study suggests that after 1 week challenged by *A. typica*, there is reduce of naive Tregs population. The previous study reported that *A. simplex* challenge represents the balanced between Th_1_/Th_2_ responses [13]. IL-4 secretion triggers IgE production by B-lymphocyte, while IL-5 involved in eosinophilic production under anisakiasis [26,27]. Elevated Th_1_-mediated response during anisakiasis was associated with Th_17_ activation [28] and severe GI symptoms, which displays the clinical manifestation in patients [29]. Furthermore, one of DCs subset (CD11c^mid^CD45RB^high^) reported to activate CD4^+^ T cells to secrete the high levels of both IFN-γ and IL-4 in nematode-infected mice [30].

## Conclusion

We have evaluated the immune profile after *A. typica* challenged, which have generated new possibilities to understand the role of *A. typica* after infected mice. In our present study, *A. typica* infection exhibits a mixed Th_1_/Th_2_ pattern which more skewed to the pro-inflammatory state than the classical model of an allergic reaction to food antigens. Further studies are required to understanding the molecular mechanism of *A. typica* infection which may imply the human allergic reaction during parasite infection. Further experiments are needed to explain the detailed mechanism of *Anisakis* infection. More appropriate experiments such as different route of administration and sample preparation are expected to complete the detailed *Anisakis* infection mechanism.

## Authors’ Contributions

Concept: AA, ES, and AMH; Design: LH; Supervision: AA, ES, and AMH; Resources: LH; Materials: LH; Data collection and/or processing: LH; Analysis and/or interpretation: LH, AA, ES, and AMH; Literature search: LH; Writing manuscript: LH; Critical review: AA, ES, and AMH. All authors read and approved the final manuscript.
